# Prognostic value of ^18^F-FDG PET in uterine cervical cancer patients with stage IIICr allocated by imaging

**DOI:** 10.1038/s41598-023-46261-2

**Published:** 2023-11-01

**Authors:** Yuzu Isaji, Hideaki Tsuyoshi, Tetsuya Tsujikawa, Makoto Orisaka, Hidehiko Okazawa, Yoshio Yoshida

**Affiliations:** 1 Department of Obstetrics and Gynecology, University of Fukui, 23-3 Matsuoka-Shimoaizuki, Eiheiji-cho, Yoshida-gun, Fukui, 910-1193 Japan; 2 Department of Radiology, University of Fukui, Fukui, Japan; 3 Biomedical Imaging Research Center, University of Fukui, Fukui, Japan

**Keywords:** Cancer, Oncology

## Abstract

The effect on survival of radiographic lymph node metastasis in uterine cervical cancer patients is more important than before, even though its prognostic value not been well investigated. The aim of our study is to evaluate the prognostic potential of ^18^F-fluorodeoxyglucose Positron Emission Tomography (^18^F-FDG PET) compared with Computed Tomography (CT) in uterine cervical cancer patients with stage IIICr allocated by imaging. Fifty-five patients with biopsy-proven primary cervical cancer underwent definitive radiation therapy for stages IIB–IVB of The International Federation of Gynecology and Obstetrics (FIGO) 2018 classifications. The prognostic performance of pretreatment ^18^F-FDG PET and CT for assessing lymph node metastasis was evaluated by two experienced readers. The PET and CT findings were correlated with the risk of progression-free survival (PFS) and overall survival (OS). Kaplan–Meier survival curves showed that PFS was significantly worse in patients with positive lymph nodes on ^18^F-FDG PET than in those patients with negative lymph nodes on ^18^F-FDG PET (p = 0.003), whereas there was no significant difference in PFS between patients with lymph nodes sized ≥ 1 cm and those sized < 1 cm (p = 0.140). Univariate analysis showed that positive lymph nodes on ^18^F-FDG PET was significantly associated with poor PFS (p = 0.006), whereas lymph node size was not significantly associated with poor PFS (p = 0.145). In multivariate analysis, positive lymph nodes on ^18^F-FDG PET was significantly associated with poor PFS (p = 0.006) and was an independent prognostic factor for PFS. ^18^F-FDG PET offers high prognostic value for patients with stage IIICr allocated by imaging compared with CT, suggesting that ^18^F-FDG PET might be useful in clinical staging decisions and thus promote optimal diagnostic and therapeutic strategies.

## Introduction

Cervical cancer is the fourth most common cancer in women worldwide, with more than half a million cases diagnosed and 0.3 million deaths annually^1^. Until recently, clinical staging of cervical cancer was based on the 2009 FIGO (The International Federation of Gynecology and Obstetrics) classifications^2^. However, this system lacked descriptions of imaging findings other than hydronephrosis and distant metastasis, and did not take into account lymph node involvement, which is associated with a poor prognosis^3^. The 2018 FIGO system highlighted the utility of imaging and permitted its use, when available, as part of clinical staging^4,5^. The revised system better recognizes the risk of metastasis and recurrence by the addition of tumor size subdivisions (IB1, IB2, and IB3) and by taking into account the status of regional lymph nodes detected either radiographically or pathologically (IIIC1 and IIIC2). The notations of r (imaging) and p (pathology) indicate the findings used to allocate a case to stage IIIC. Therefore, the revised system has afforded a greater importance to imaging findings in the planning of optimal treatment, although pathological findings supersede imaging findings.

In the FIGO 2018 system, the choice of imaging modality for nodal evaluation is not specified, and instead depends upon the availability of imaging modalities and patients’ ability to access these, taking into account affordability. In addition, the revised FIGO system does not provide imaging criteria for discriminating between malignancy and inflammation/infection, which is left to the discretion of the clinician. CT (Computed Tomography) has been widely used for this purpose because of its cost- and time-effectiveness. In a meta-analysis, ^18^F-fluorodeoxyglucose (^18^F-FDG) PET (Positron Emission Tomography) or PET/CT demonstrated higher sensitivity and comparable specificity to MRI (Magnetic Resonance Imaging) and CT^6^. However, a more recent meta-analysis has reported comparable performances of CT, MRI, and PET for assessing nodal metastasis, with low sensitivity but high specificity^7^. Accordingly, the optimal imaging modalities for detecting lymph node metastasis in cervical cancers remain unclear, although ^18^F-FDG PET may be a valid alternative.

Compared with the FIGO 2009 system, the FIGO 2018 system provides improved discriminatory ability to predict survival, particularly in patients with stage IB tumors. In patients with stage IIIC tumors, however, its impact on survival varies depending on local tumor factors because of controversy regarding treatment choices among T stages^8–10^. Moreover, among patients with stage IIIC tumors, the treatment choices might include surgery, radiation therapy, and chemotherapy, which increases the difficulty of accurately predicting prognosis. With the revised 2018 staging, patients with stage IIIC disease are more likely to receive radiotherapy-based treatment. Accordingly, more accurate biomarkers are required for predicting survival, particularly for stage IIICr, for which the diagnosis is image based.

Hence, the aim of the present study was to evaluate the prognostic utility of CT and ^18^F-FDG PET for patients with stage IIICr uterine cervical cancer who received definitive radiotherapy. To the best of our knowledge, this study is the first to demonstrate that compared with CT, ^18^F-FDG PET offers higher prognostic value for patients with stage IIICr, and indicates that ^18^F-FDG PET might assist in deciding the optimal diagnostic and therapeutic strategy based on clinical staging.

## Materials and methods

### Patients

We retrospectively reviewed the medical records of 55 patients (mean age, 61.4 years; age range, 37–86 years) with biopsy-proven primary cervical cancer who were treated between November 2006 and November 2021. The inclusion criteria were as follows: ^18^F-FDG PET/CT or ^18^F-FDG PET/MRI as well as CT, performed for initial staging based on the Japanese Imaging Guidelines 2021 of the Japan Radiological Society (http://www.radiology.jp/english/guideline.html); and definitive radiotherapy at our institution for stages IIB–IVB of the FIGO 2018 classifications, and higher than T2b based on the Japanese Guidelines of the Japan Society of Gynecologic Oncology. The exclusion criteria were stage T1a–T2a of the Japanese Guidelines even if staged as IIIC of the FIGO 2018 classifications, previous definitive surgery or systemic chemotherapy, the pre-therapeutic surgical assessment of para-aortic lymph node status, and palliative radiotherapy because of other factors affecting the prognosis. All ^18^F-FDG PET/CT, PET/MRI, and CT examinations were performed within 2 months prior to treatment. The maximum interval between the ^18^F-FDG PET/CT or PET/MRI and the CT examinations was 21 days (mean, 6.5 days; range, 1–21 days). In this study, the imaging examinations of 15 of the 55 patients who underwent CT and 10 of the 38 patients who underwent ^18^F-FDG PET/CT were performed at other institutions. All 17 ^18^F-FDG PET/MRI examinations were performed at our institution. The study protocol was approved by the institutional review board of the University of Fukui Hospital (No. 20210730). All methods were performed in accordance with the approved guidelines and regulations.

### ^18^F-FDG PET/CT

^18^F-FDG PET was performed with a combined PET/CT scanner (Discovery LS; GE Healthcare, Milwaukee, WI) used mainly for clinical purposes. The PET/CT scanner incorporates an integrated four-slice multidetector CT scanner used for attenuation correction. The CT scanning parameters were as follows: auto mA (upper limit, 40 mA; noise index, 20), 140 kVp, 5-mm section thickness, 15-mm table feed, and pitch of 4. After fasting for at least 4 h, patients received intravenous injection of 185 MBq of ^18^F-FDG and image acquisition began 50 min after the injection. Whole-body emission scanning was performed from the head to the inguinal region with 2 min per bed position (seven to eight bed positions). PET data were reconstructed by ordered subset expectation maximization (OSEM), selecting 14 subsets and 2 iterations, a 128 × 128 matrix, voxel size (width, length and height) = 4 × 4 × 4.25 mm, and post-smoothing with an 8-mm Gaussian filter. The reconstructed images were then converted to semi-quantitative images corrected by the injection dose and subject’s body weight (= standardized uptake value: SUV)^11^.

### ^18^F-FDG PET/MRI

#### Whole-body PET/MRI

Patients fasted for at least 4 h prior to intravenous injection of 200 MBq of 18F-FDG. Fifty minutes after injection, patients were transferred to a whole-body 3.0-T PET/MR scanner (Signa PET/MR; GE Healthcare, Waukesha, WI). Anatomical coverage was from the vertex to the mid-thigh. PET acquisition was performed in three-dimensional (3D) mode with 5.5 min/bed position (89 slices/bed) in 5–6 bed positions with a 24-slice overlap. A two-point Dixon 3D volumetric interpolated T1-weighted fast spoiled gradient echo sequence was acquired at each table position and was used to generate MR attenuation correction (MR-AC) maps. Dixon-based MR-AC classifies body tissues into soft tissue, fat, and air. PET data were reconstructed by OSEM, selecting 14 subsets and 3 iterations, and post-smoothing with a 3-mm Gaussian filter^12^.

#### Pelvic PET/MRI

After whole-body scanning and a brief break for urination, the patient was repositioned in the PET/MR scanner. The pelvic PET scan was performed as a 3D acquisition in list mode with 15 min/bed position (89 slices/bed) in 1–2 bed positions with a 24-slice overlap. Regional PET data were reconstructed with OSEM, selecting 16 subsets and 4 iterations, and post-smoothing with a 4-mm Gaussian filter. The reconstructed images were then converted to SUV images. For pelvic MRI, T2-weighted images were acquired in the sagittal, transaxial, and coronal planes, using the following image parameters: TR, 4000–7000 ms; TE, 146 ms; section thickness, 4 mm; section overlap, 0 mm; flip angle, 100°; FOV, 240 × 240 mm; matrix, 384 × 384; two excitations; and bandwidth, 83.3 kHz^12^.

### CT

CT examinations covering the chest, abdomen, and pelvis were performed using a 64-slice multidetector CT scanner (Discovery CT 750HD; GE Medical Systems, Milwaukee, WI)^12^.

### Image interpretation

Images were analyzed on a dedicated workstation (Advantage Workstation 4.6; GE Healthcare). Two board-certificated radiologists/nuclear medicine physicians, each with double certifications and specializing in gynecological imaging, evaluated the ^18^F-FDG PET/CT or PET/MRI, and the CT images retrospectively and reached consensus decisions. Images were evaluated for pelvic lymph node metastasis (IIIC1r) and para-aortic lymph node metastasis (IIIC2r). Both readers were blinded to the results of the other imaging studies, histopathological findings, and clinical data. Each dataset was reviewed as the consensus decision of the two readers after a minimum interval of 3 weeks to avoid any decision threshold bias due to reading-order effects. For CT interpretation, several previous standard criteria related to nodal metastatic staging of cervical cancer were used as the reference criteria^7,13^. Lymph nodes larger than 1 cm in short-axis diameter were graded as malignant. In the ^18^F-FDG PET/CT and PET/MRI interpretations, classification of lymph nodes as cancer-positive was based on the presence of focally appreciable metabolic activity above that of normal muscle; or as asymmetric metabolic activity greater than that of normal-appearing lymph nodes at the same level in the contralateral pelvis, in a location corresponding to the lymph node chains on CT images, with reference to previous reports^7,12,14^.

### Reference standard

Because clinical and ethical standards of patient management do not require surgery or sampling of all detected lesions, a modified reference standard was used for lesions without histopathological sampling to take into account all prior and follow-up imaging. A decrease in size and/or SUVmax under therapy or an increase in size and/or SUVmax without therapy was regarded to indicate malignancy. PET-negative and inconspicuous lesions with constant size were rated as benign.

### Endpoints

The primary endpoints were progression-free survival (PFS) and overall survival (OS). The date on which definitive radiotherapy was performed was used as the starting point for PFS and OS. Tumor progression was confirmed by either imaging or tissue biopsy showing evidence of progressive disease according to the World Health Organization Response Evaluation Criteria In Solid Tumors (RECIST) guidelines (version 1.1). The secondary endpoint was the relevance of clinicopathological parameters (histology, and parametrial invasion extending to the pelvic wall) and combined treatment (concurrent chemoradiotherapy and brachytherapy) to the PET and CT findings.

### Statistical analysis

All data were collected in a structured database and analyzed using SPSS statistics, version 24 (IBM). Pearson's Chi-square test was used to analyze relationships between clinical characteristics and the PET and CT findings. The Kaplan–Meier method was used to estimate PFS and OS, and these were compared using the log-rank test. Cox proportional hazards regression modeling was used for univariate and multivariate analyses. Significance was defined as a P level of less than 0.05 (2-sided testing).

### Ethics approval and consent to participate

The study protocol has been approved by the institutional review board of the University of Fukui Hospital (No. 20210730). This study is a retrospective study. Patients provided written informed consent to participate in the study and anonymous clinical data were used. Patients provided written consent for the use of residual samples and were offered opt-out as disclosed on the website (https://www.u-fukui.ac.jp/cont_about/disclosure/privacy/).

## Results

### Patients

**Figure 1:**
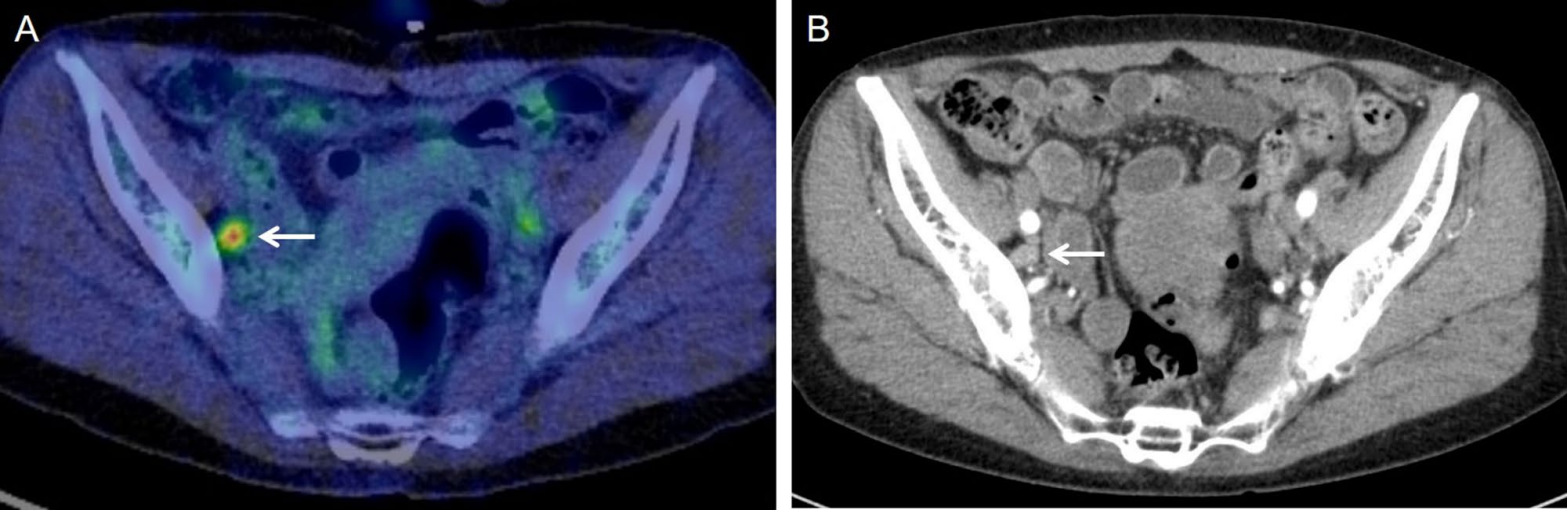
A 66-year-old woman with stage IIIC1 cervical squamous cell cancer. (**A**) ^18^F-FDG PET/CT image shows positive lymph nodes on ^18^F-FDG PET in a right pelvic lymph node (arrow). (**B**) CT image shows the lymph node (arrow) of size 0.78 cm (< 1 cm).

**Figure 2:**
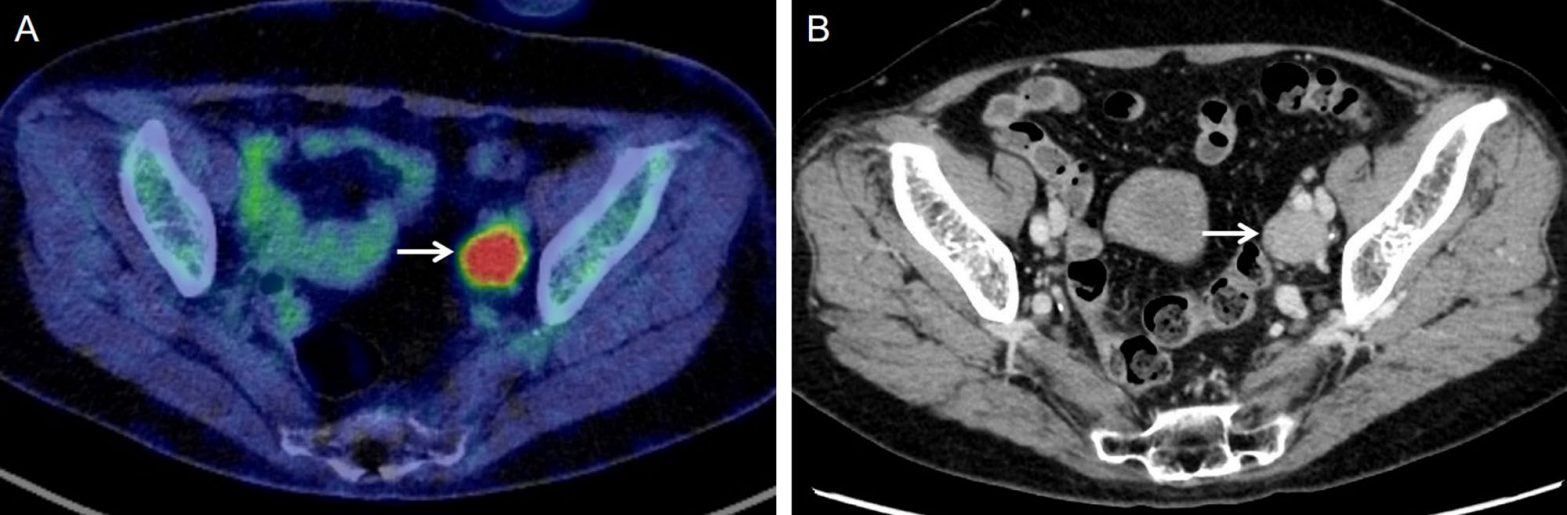
A 72-year-old woman with stage IIIC1 cervical squamous cell cancer. (**A**) ^18^F-FDG PET/CT image shows positive lymph nodes on ^18^F-FDG PET in a left pelvic lymph node (arrow). (**B**) CT image shows the lymph node (arrow) of size 2.08 cm (≥ 1 cm).

Table 1 lists the clinical information of the 55 patients included in the study. According to the FIGO 2018 criteria^4,5^, stage was classified as IIB in 1, IIIA in 2, IIIB in 12, IIIC1r in 17, IIIC2r in 12, IVA in 4, and IVB in 7 patients. The histopathologic types of the primary tumors were squamous cell carcinoma (n = 51) and adenocarcinoma (n = 4). All 55 patients had parametrial invasion, which extended to the pelvic wall in 42. Of the total patients, 36 patients received concurrent chemoradiotherapy with weekly cisplatin (mean, 5.2 times; range, 1–8 times) and 37 received combined brachytherapy (mean, 3.4 times; range, 2–5 times). Regarding imaging modality, 17 patients underwent PET/MRI and 38 patients underwent PET/CT. In the PET and CT findings of lymph nodes in the overall patients, 18 patients showed negative lymph nodes on ^18^F-FDG PET and size < 1 cm, 11 showed positive lymph nodes on ^18^F-FDG PET and size < 1 cm, 0 showed negative lymph nodes on ^18^F-FDG PET and size ≥ 1 cm, and 26 showed positive lymph nodes on ^18^F-FDG PET and size ≥ 1 cm (Supplemental Table 1). The median follow-up period was 34 months (range, 6.7–158.4 months). Figure 1 shows a representative case of ^18^F-FDG PET/CT and CT with positive lymph nodes on ^18^F-FDG PET and size < 1 cm, whereas Fig. 2 shows that of ^18^F-FDG PET/CT and CT with positive lymph nodes on ^18^F-FDG PET and size ≥ 1 cm. Tumor progression occurred in 31 patients (56.4%) during the follow-up period (IIB in 0, IIIA in 0, IIIB in 2, IIIC1r in 11, IIIC2r in 9, IVA in 3, and IVB in 6 patients), and 26 patients (47.3%) died (IIB in 0, IIIA in 1, IIIB in 3, IIIC1r in 6, IIIC2r in 9, IVA in 3, and IVB in 4 patients) (Supplemental Table 1).

**Table 1:** Patient and tumor characteristics.

Characteristic	n	%
Total number of patients	55	100
FIGO stage		
IIB	1	1.8
IIIA	2	3.6
IIIB	12	21.8
IIIC1r	17	30.9
IIIC2r	12	21.8
IVA	4	7.3
IVB	7	12.7
Histopathologic type		
Squamous cell carcinoma	51	92.7
Adenocarcinoma	4	7.3
Parametrial invasion extending to pelvic wall		
Absent	13	23.6
Present	42	76.4
Combined chemotherapy		
Radiotherapy only	19	34.5
Concurrent chemoradiotherapy	36	65.5
Combined brachytherapy		
External beam radiation only	18	32.7
Combined brachytherapy	37	67.3
Modality combined with PET		
PET/MRI	17	30.9
PET/CT	38	69.1
Imaging findings of lymph nodes		
Negative lymph nodes on ^18^F-FDG PET and size < 1 cm	18	32.7
Positive lymph nodes on ^18^F-FDG PET and size < 1 cm	11	20.0
Negative lymph nodes on ^18^F-FDG PET and size ≥ 1 cm	0	0
Positive lymph nodes on ^18^F-FDG PET and size ≥ 1 cm	26	47.3
Tumor progression		
Absent	24	43.6
Present	31	56.4
Survival		
Alive	29	52.7
Deceased	26	47.3

*FIGO* The International Federation of Gynecology and Obstetrics, *PET* Positron Emission Tomography, *MRI* Magnetic Resonance Imaging, *CT* Computed Tomography, ^*18*^*F-FDG* ^18^F-fluorodeoxyglucose.

### Correlation of imaging findings with clinicopathological parameters and combined treatments

Table 2 lists the clinicopathological parameters according to the PET and CT findings. There was no significant correlation of histopathological subtype with positive lymph nodes on ^18^F-FDG PET or lymph node size (p = 0.430 and p = 0.652, respectively); or of parametrial invasion extending to the pelvic wall with positive lymph nodes on ^18^F-FDG PET or lymph node size (p = 0.259 and p = 0.410, respectively). Combined therapy with concurrent chemoradiotherapy was not significantly correlated with positive lymph nodes on ^18^F-FDG PET or lymph node size (p = 0.125 and p = 0.607, respectively). Combined therapy with brachytherapy was also not significantly correlated with positive lymph nodes on ^18^F-FDG PET or lymph node size (p = 0.336 and p = 0.284, respectively). In terms of modality, the use of PET/MRI or PET/CT was not significantly correlated with positive lymph nodes on ^18^F-FDG PET or lymph node size (p = 0.070 and p = 0.393, respectively). Positive lymph nodes on ^18^F-FDG PET was significantly correlated with tumor progression (p = 0.001) but lymph node size was not (p = 0.060). There was no significant correlation of positive lymph nodes on ^18^F-FDG PET or lymph node size with death (p = 0.200 and p = 0.455, respectively). There was a significant correlation between positive lymph nodes on ^18^F-FDG PET and lymph node size (p < 0.001).

**Table 2:** Correlation of imaging findings with clinicopathological parameters.

	Lymph nodes on ^18^F-FDG PET		P	CT		P
	Positive	Negative		Size ≥ 1 cm	Size < 1 cm	
Total number of patients	37	18		26	29	
Histopathologic type			0.430			0.652
Squamous cell carcinoma	35	16		24	27	
Adenocarcinoma	2	2		2	2	
Parametrial invasion extending to pelvic wall			0.259			0.410
Absent	10	3		7	6	
Present	27	15		19	23	
Combined chemotherapy			0.125			0.607
Radiotherapy only	11	8		9	10	
Concurrent chemoradiotherapy	26	10		17	19	
Combined brachytherapy			0.336			0.284
External beam radiation only	14	4		10	8	
Combined brachytherapy	23	14		16	21	
Modality combined with PET			0.070			0.393
PET/MRI	14	3		9	8	
PET/CT	23	15		17	21	
Imaging findings of lymph nodes			< 0.001*			< 0.001*
Negative lymph nodes on ^18^F-FDG PET and size < 1 cm	0	18		0	18	
Positive lymph nodes on ^18^F-FDG PET and size < 1 cm	11	0		0	11	
Negative lymph nodes on ^18^F-FDG PET and size ≥ 1 cm	0	0		0	0	
Positive lymph nodes on ^18^F-FDG PET and size ≥ 1 cm	26	0		26	0	
Tumor progression			0.001*			0.060
Absent	10	14		8	16	
Present	27	4		18	13	
Survival			0.200			0.455
Alive	17	12		13	16	
Deceased	20	6		13	13	

*Pearson's Chi-square test.

*PET* Positron Emission Tomography, *MRI* Magnetic Resonance Imaging, *CT* Computed Tomography, ^*18*^*F-FDG* ^18^F-fluorodeoxyglucose.

### Prognostic effect of positive lymph nodes on ^18^F-FDG PET and lymph node size

**Figure 3:**
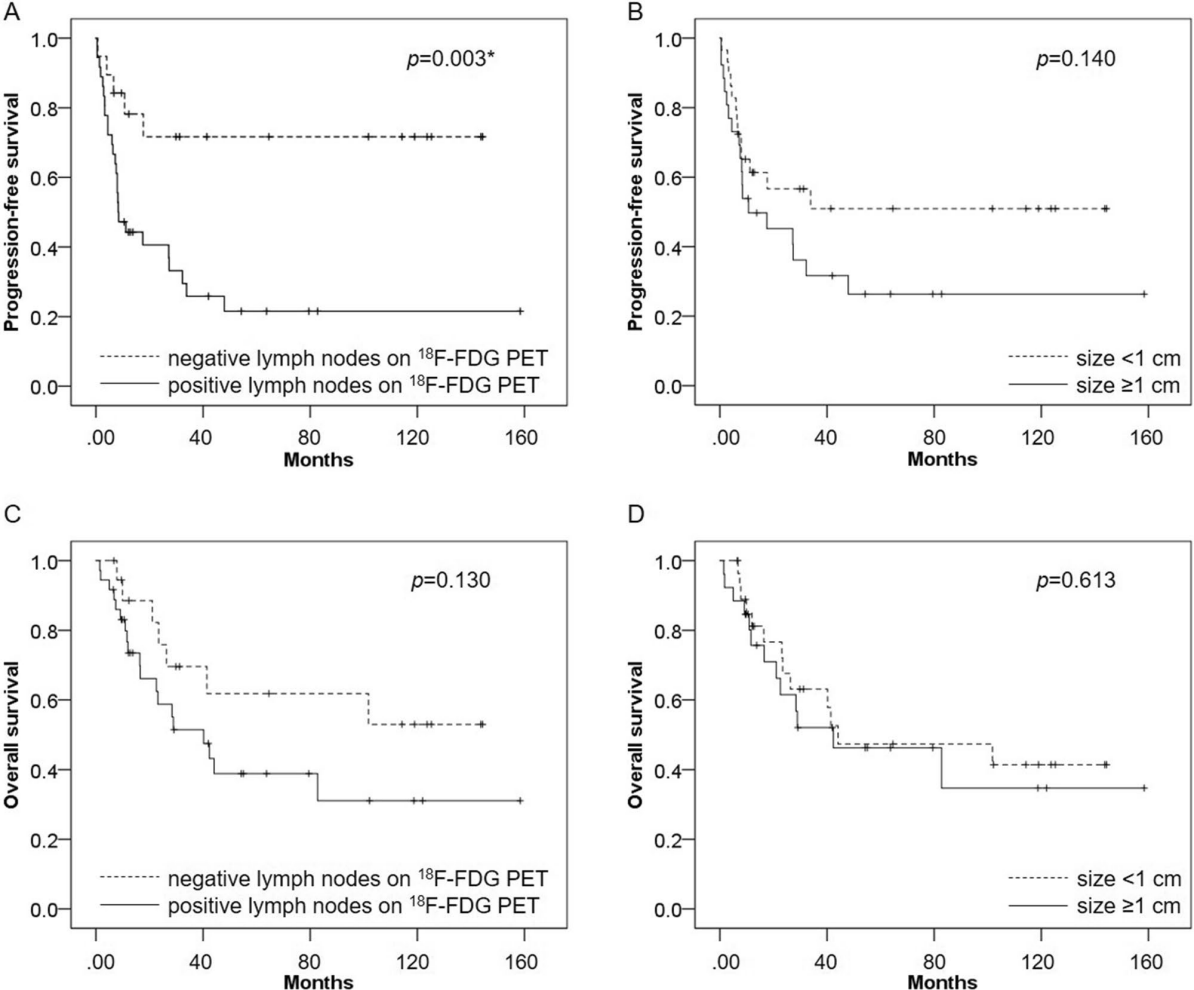
Kaplan–Meier survival curves for PFS and OS among patients with uterine cervical cancer who underwent ^18^F-FDG PET/CT or PET/MRI, and CT before definitive radiation therapy. (**A**) PFS was significantly worse in patients with positive lymph nodes on ^18^F-FDG PET than in those with negative lymph nodes on ^18^F-FDG PET (p = 0.003). (**B**) There was no significant difference in PFS between patients with lymph node size ≥ 1 cm and those with size < 1 cm (p = 0.140). (**C**) There was no significant difference in OS between patients with positive lymph nodes on ^18^F-FDG PET and those with negative lymph nodes on ^18^F-FDG PET (p = 0.130). (**D**) There was no significant difference in OS between patients with lymph node size ≥ 1 cm and those with size < 1 cm (p = 0.613).

In all 55 patients, Kaplan–Meier survival curves showed significantly worse PFS in patients with positive lymph nodes on ^18^F-FDG PET than in those with negative lymph nodes on ^18^F-FDG PET (p = 0.003), whereas there was no significant difference in PFS between patients with lymph node size ≥ 1 cm and those with size < 1 cm (p = 0.140). In terms of OS, there was no statistically significant difference between positive lymph nodes on ^18^F-FDG PET and lymph nodes ≥ 1 cm (p = 0.130 and p = 0.613, respectively) (Fig. 3).

In 32 T3b patients with IIIB in 12 or IIICr in 20, Kaplan–Meier survival curves showed significantly worse PFS in patients with positive lymph nodes on ^18^F-FDG PET than in those with negative lymph nodes on ^18^F-FDG PET (p = 0.005), whereas there was no significant difference in PFS between patients with lymph node size ≥ 1 cm and those with size < 1 cm (p = 0.336). In terms of OS, there was no statistically significant difference between positive lymph nodes on ^18^F-FDG PET and lymph nodes ≥ 1 cm (p = 0.129 and p = 0.953, respectively) (Supplemental Fig. 1).

In 24 T3b patients with IIIB in 12 or IIICr1 in 12, Kaplan–Meier survival curves showed the trend of worse PFS in patients with positive lymph nodes on ^18^F-FDG PET than in those with negative lymph nodes on ^18^F-FDG PET (p = 0.062), whereas there was no significant difference in PFS between patients with lymph node size ≥ 1 cm and those with size < 1 cm (p = 0.834). In terms of OS, there was no statistically significant difference between positive lymph nodes on ^18^F-FDG PET and lymph nodes ≥ 1 cm (p = 0.775 and p = 0.425, respectively) (Supplemental Fig. 2).

In 20 T3b patients with IIIB in 12 or IIICr2 in 8, Kaplan–Meier survival curves showed significantly worse PFS in patients with positive lymph nodes on ^18^F-FDG PET than in those with negative lymph nodes on ^18^F-FDG PET (p < 0.001). Kaplan–Meier survival curves also showed significantly worse PFS in patients with lymph node size ≥ 1 cm than in those with size < 1 cm (p < 0.001). In terms of OS, there was statistically significant difference between both positive lymph nodes on ^18^F-FDG PET and lymph nodes ≥ 1 cm (p = 0.002 and p = 0.002, respectively) (Supplemental Fig. 3).

**Figure 4:**
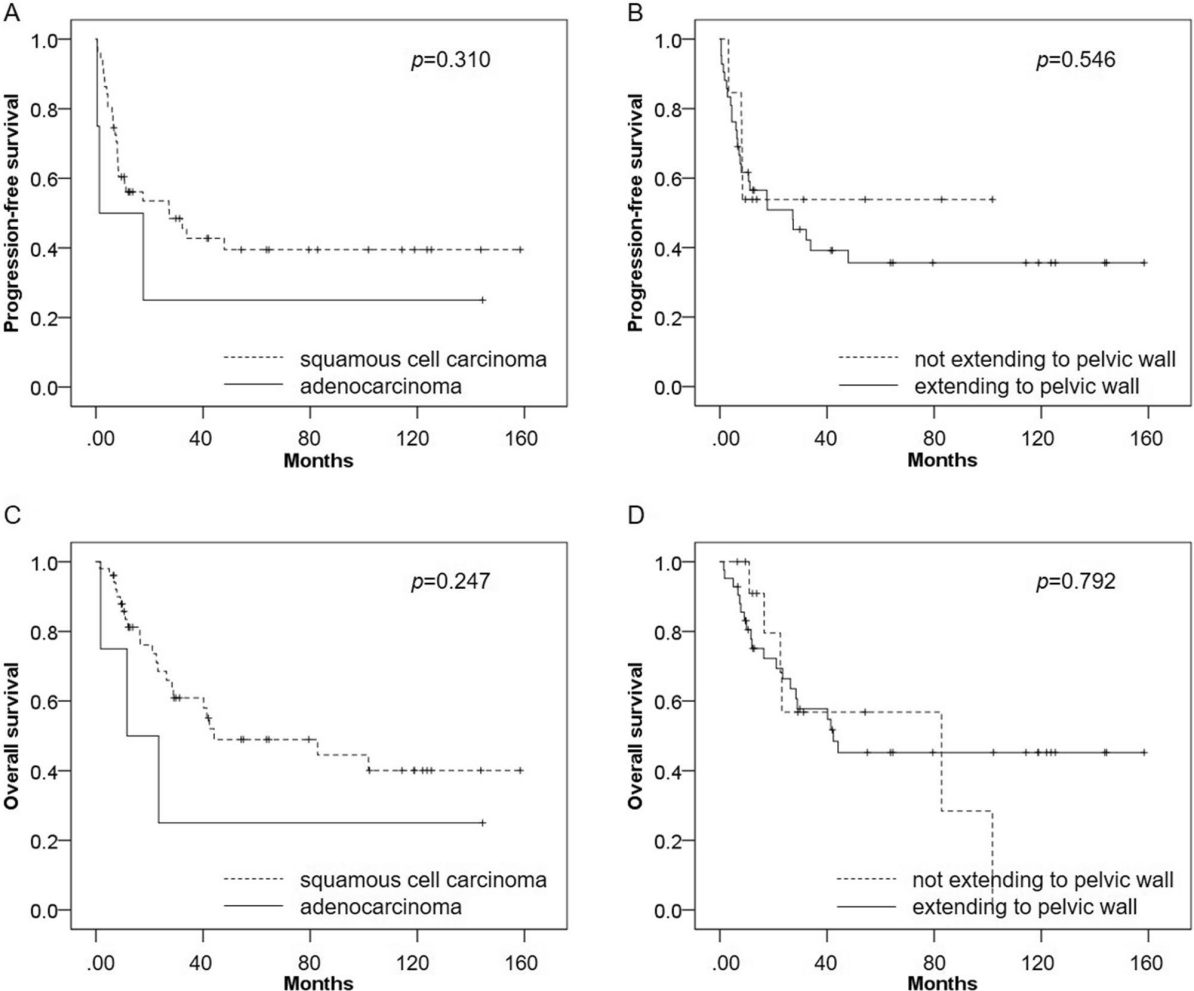
(**A**) In terms of clinicopathological parameters, there was no significant difference in PFS between patients with adenocarcinoma and those with squamous cell carcinoma (p = 0.310). (**B**) There was no significant difference in PFS between patients who had parametrial invasion extending to the pelvic wall and those who did not (p = 0.546). (**C**) There was no significant difference in OS (p = 0.247) between patients with adenocarcinoma and those with squamous cell carcinoma. (**D**) There was no significant difference in OS between patients who had parametrial invasion extending to the pelvic wall and those who did not (p = 0.792).

In terms of clinicopathological parameters, there was no significant difference in PFS or OS between patients with adenocarcinoma and those with squamous cell carcinoma (p = 0.310 and p = 0.247, respectively). There was no significant difference in PFS or OS between patients with parametrial invasion extending to the pelvic wall and those who did not (p = 0.546 and p = 0.792, respectively) (Fig. 4).

**Figure 5:**
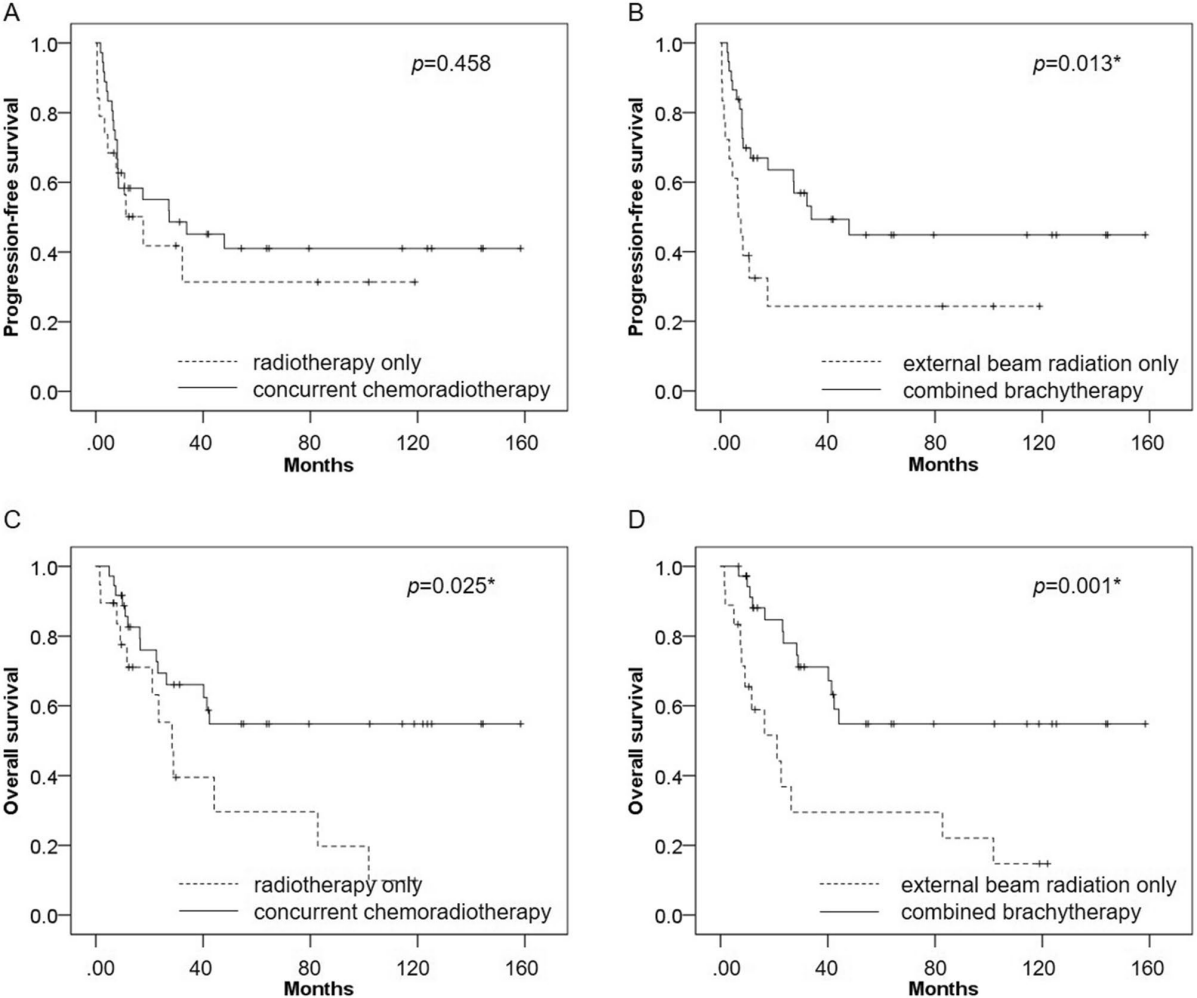
(**A**) In terms of combined treatments, there was no significant difference in PFS between patients treated with concurrent chemoradiotherapy and those treated with radiotherapy alone (p = 0.458). (**B**) PFS was significantly better in patients treated with external beam radiation combined with brachytherapy than in those treated with external beam radiation alone (p = 0.013). (**C**) OS was significantly better patients treated with concurrent chemoradiotherapy than in those treated with radiotherapy alone (p = 0.025). (**D**) OS was significantly better in patients treated with external beam radiation combined with brachytherapy than in those treated with external beam radiation alone (p = 0.001).

In terms of combined treatments, there was no significant difference in PFS between patients treated with concurrent chemoradiotherapy and those treated with radiotherapy alone (p = 0.458), whereas OS was significantly better in patients treated with concurrent chemoradiotherapy than in those treated with radiotherapy alone (p = 0.025). PFS and OS were significantly better in patients treated with external beam radiation combined with brachytherapy than in those treated with external beam radiation alone (p = 0.013 and p = 0.001, respectively) (Fig. 5).

Regarding PFS, univariate analysis showed that positive lymph nodes on ^18^F-FDG PET was significantly associated with poor PFS (p = 0.006), whereas lymph node size was not (p = 0.145). Combined brachytherapy was also significantly associated with PFS (p = 0.017). In multivariate analysis, positive lymph nodes on ^18^F-FDG PET and combined brachytherapy were both significantly associated with PFS (p = 0.006 and p = 0.020, respectively) and were independent prognostic factors for PFS (Table 3).

**Table 3:** Prognostic factors for progression-free survival.

Variable	Univariate analysis	P	Multivariate analysis	P
	Hazard ratio (95% CI)		Hazard ratio (95% CI)	
Age (y)	1.00 (0.97–1.03)	0.969		
Histopathologic type (adenocarcinoma)	1.84 (0.56–6.06)	0.318		
Parametrial invasion extending to pelvic wall	1.32 (0.54–3.21)	0.548		
Concurrent chemoradiotherapy	0.75 (0.36–1.58)	0.461		
Brachytherapy	0.41 (0.20–0.55)	0.017*	0.42 (0.20–0.87)	0.020*
Positive lymph nodes on ^18^F-FDG PET	3.86 (1.48–10.10)	0.006*	3.82 (1.46–10.03)	0.006*
Size ≥ 1 cm	1.70 (0.83–3.47)	0.145		

*Cox’s uni- and multivariate analysis.

^*18*^*F-FDG PET* ^18^F-fluorodeoxyglucose Positron Emission Tomography.

Regarding OS, univariate analysis showed that concurrent chemoradiotherapy and combined brachytherapy were significantly associated with OS (p = 0.030 and 0.002, respectively). Neither positive lymph nodes on ^18^F-FDG PET nor lymph node size was significantly associated with poor OS (p = 0.136 and 0.613, respectively). In multivariate analysis, no independent prognostic factor for OS was identified other than combined brachytherapy (p = 0.020) (Table 4).

**Table 4:** Prognostic factors for overall survival.

Variable	Univariate analysis	P	Multivariate analysis	P
	Hazard ratio (95% CI)		Hazard ratio (95% CI)	
Age (y)	1.01 (0.98–1.44)	0.47		
Histopathologic type (adenocarcinoma)	2.01 (0.60–6.72)	0.257		
Parametrial invasion extending to pelvic wall	0.88 (0.35–2.21)	0.792		
Concurrent chemoradiotherapy	0.43 (0.20–0.92)	0.030*	0.65 (0.28–1.54)	0.330
Brachytherapy	0.30 (0.14–0.65)	0.002*	0.36 (0.15–0.86)	0.020*
Positive lymph nodes on ^18^F-FDG PET	1.94 (0.81–4.66)	0.136		
Size ≥ 1 cm	1.22 (0.56–2.69)	0.613		

*Cox’s uni- and multivariate analysis.

^*18*^*F-FDG PET* ^18^F-fluorodeoxyglucose Positron Emission Tomography.

In 32 T3b patients with IIIB in 12 or IIICr in 20, Regarding PFS, univariate analysis showed that positive lymph nodes on ^18^F-FDG PET was significantly associated with poor PFS (p = 0.014), whereas lymph node size was not (p = 0.342). In multivariate analysis, positive lymph nodes on ^18^F-FDG PET and combined brachytherapy were both significantly associated with PFS (p = 0.010 and p = 0.032, respectively) and were independent prognostic factors for PFS (Supplemental Table 2).

Regarding OS, univariate analysis showed that brachytherapy was significantly associated with OS (p = 0.019). Neither positive lymph nodes on ^18^F-FDG PET nor lymph node size was significantly associated with poor OS (p = 0.143 and 0.954, respectively). In multivariate analysis, no independent prognostic factor for OS was identified other than combined brachytherapy (p = 0.011) (Supplemental Table 3).

## Discussion

To the best of our knowledge, this is the first study to investigate the prognostic value of ^18^F-FDG PET for patients with stage IIICr cervical cancer based on the revised 2018 FIGO staging system in comparison with conventional staging by CT. PFS was significantly worse in patients with positive lymph nodes on ^18^F-FDG PET than in patients with negative lymph nodes on ^18^F-FDG PET, whereas there was no significant difference in PFS between patients with lymph nodes sized ≥ 1 cm compared with those sized < 1 cm. Univariate and multivariate analysis showed that positive lymph nodes on ^18^F-FDG PET was significantly associated with poor PFS and was an independent prognostic factor for PFS. These findings suggest that ^18^F-FDG PET might be useful in clinical staging decisions and thus promote optimal diagnostic and therapeutic strategies.

The 2018 FIGO staging system includes lymph node metastasis as stage IIIC and recommends the use of imaging findings as well as pathological analysis in identification of lymph node metastases. It has been reported that stage IIIC disease negatively affected prognosis in cervical cancer^8^. However, the impact on survival varies with T stage, and can be also affected by treatment including surgery, radiotherapy, and chemotherapy. Numerous studies have reported the prognostic value of pathologically proven lymph node metastasis by surgery (IIICp). In analysis of pathologically positive nodes based on The National Cancer Databases, IIIC2 patients with para-aortic lymph node metastasis showed a worse 5-year survival rate (45.1%) than that of IIIC1 patients with pelvic lymph node metastasis (70.9%), which indicates that the location of lymph node metastasis is a prognostic factor^9^. In terms of the impact of the size of pathologic lymph node metastasis on survival, diameter ≥ 20 mm of surgically proven lymph node metastasis has also been associated with poor prognosis^15^. Lymph node ratio, as the percentage of metastatic lymph nodes to total lymph nodes recovered, is a newly emerging prognostic factor in cervical cancer^16^. Many studies have reported the accuracy of pathologically confirmed lymph node metastasis as a prognostic biomarker. However, following the 2018 revision of FIGO staging, patients with stage IIIC are more likely to receive radiotherapy-based treatment even if the T stage is relatively early, which suggests that the effect on survival of radiographic lymph node metastasis is more important than before, even though its prognostic value not been well investigated.

Regarding the prognostic value of the size of lymph node metastasis measured by imaging modalities, Wang et al. have reported that in patients treated with definitive radiotherapy, the presence of three or more lymph nodes ≥ 1 cm in short axis diameter measured by CT or MRI was an independent prognostic factor for overall survival^17^. Song et al. have reported that lymphadenopathy with diameter ≥ 15 mm showed much worse overall survival compared with no lymphadenopathy and with lymphadenopathy < 15 mm^18^, which suggests that radiographic lymphadenopathy might be a prognostic biomarker in cervical cancer. The prognostic value of ^18^F-FDG PET has been also studied. Kidd et al. have shown that patients with PET-positive lymph nodes had significantly worse disease-specific survival than those with PET-negative lymph nodes, although this study included patients who received various treatments, such as surgery, surgery plus postoperative radiotherapy, and definitive radiotherapy^19^. De Cuypere et al. have shown that the number of ^18^F-FDG PET-positive pelvic lymph nodes was the only independent prognostic factor for OS in patients with locally advanced cervical cancer^20^. Kim et al. have shown that in patients who received definitive concurrent chemoradiotherapy, high SUV values in pelvic lymph nodes was a statistically significant prognostic factor of PFS compared with those with low SUV values^21^. This finding suggests that ^18^F-FDG PET might be also a prognostic biomarker in cervical cancer, although no previous study has compared its utility as a prognostic biomarker between size measured by imaging modalities and uptake of ^18^F-FDG PET.

In the present study, we evaluated the prognostic value of size measured by CT and positive lymph nodes on ^18^F-FDG evaluated by PET/CT or PET/MRI in cervical cancer patients staged higher than T2b who received definitive radiotherapy alone. We found that patients with positive lymph nodes on ^18^F-FDG PET had significantly worse PFS than patients with negative lymph nodes on ^18^F-FDG PET, whereas there was no significant difference in PFS between patients with lymph nodes ≥ 1 cm and those with nodes < 1 cm. The universally accepted size criterion for lymphadenopathy is a short axis diameter > 10 mm measured on imaging. However, it has been reported that more than 80% of metastatic lymph nodes are smaller than 10 mm and that more than 50% are smaller than 5 mm^22^. According to these size criteria, lymph node metastases < 10 mm are considered negative, which increases the number of false negative diagnoses. Sensitivity can be improved if a 5 mm cut-off is used, although the detection of small-sized lymph nodes might produce many false positive cases, resulting in low specificity^23^. In terms of ^18^F-FDG PET, Sironi et al. have reported that ^18^F-FDG PET/CT provided sensitivity of 100% and specificity of 99.6% for the detection of lymph nodes metastases larger than even 0.5 cm in diameter^24^. A recent meta-analysis has shown that CT, MRI, and ^18^F-FDG PET performed comparably for assessing nodal metastasis, with low sensitivity but high specificity^7^, although integrated ^18^F-FDG PET/CT demonstrated higher sensitivity and comparable specificity to MRI and CT^6,25^. Moreover, the new PET modality of ^18^F-FDG PET/MRI, which has the combined features of high soft-tissue contrast along with functional imaging of ^18^F-FDG uptake, may also have high diagnostic value in cervical cancer and potentially better diagnostic performance compared with the conventional imaging modalities of CT, MRI, and ^18^F-FDG PET/CT^12,14^. Integrated ^18^F-FDG PET/CT or PET/MRI might be superior to CT or MRI for the diagnosis of the lymph node metastasis, and thus show great promise as prognostic biomarkers.

The uptake of ^18^F-FDG in PET can be affected by physiological conditions such as inflammation and the menstrual cycle, leading to false positives. Moreover, it can be difficult to diagnose lymph node metastasis smaller than 5 mm, leading to false negatives. A meta-analysis has demonstrated that even when no suspicious para-aortic lymph node metastases were identified on ^18^F-FDG PET/CT or MRI, lymph node metastases were identified surgically in a considerable number of patients with locally advanced cervical cancer (12%, and 11%, respectively)^26^. Moreover, another meta-analysis has shown that the presence of micrometastases (0.2–2.0 mm) seems to be associated with a negative impact on both PFS and OS, and that these should be treated as macrometastases (> 2.0 mm)^27^, although the presence of micrometastases does not change the stage in FIGO 2018^4^. Accordingly, there might be limited value of these small lymph node metastases for the imaging diagnosis of lymph node metastasis (IIICr). In the present study, combined chemotherapy with definitive radiotherapy improved OS even in for patients with stage IIICr. This finding suggests that concurrent chemoradiotherapy could improve prognosis in this population, in agreement with numerous previous studies that have shown improvement of survival following concurrent chemoradiotherapy in patients with locally advanced cervical cancer^28^.

In the present study, we have also evaluated the prognostic value and relevance of clinicopathological parameters and combined treatment with the PET and CT findings. Contrary to the findings of a meta-analysis^29^, we found no significant differences in clinicopathological parameters even for risk factors that have a high impact on survival (such as adenocarcinoma and the presence of parametrial invasion extending to pelvic wall), perhaps due to the small number of patients in the present study. In terms of combined treatment, our results showed that combined brachytherapy or chemotherapy with radiotherapy improved PFS and/or OS, in agreement with previous studies^28,30^. Because the present study focused on patients with locally advanced cervical cancer staged higher than T2b, the prognostic effect of combined brachytherapy can be reasonable. In addition, as our study focused on patients with radiologically indicated and/or potential lymph node metastasis, the prognostic effect of combined chemotherapy can be reasonable and should be recommended in patients with performance status tolerable of chemotherapy.

This study had several limitations. First, we used a retrospective design, and not all examinations were performed at our institution. However, our readers re-evaluated images acquired at other hospitals and were blinded to the initial imaging findings. We also reviewed the radiologic interpretations which were performed when the patients underwent the examinations and confirm that those interpretations were consistent with this retrospective analysis. Second, the sample size was relatively small, and the long interval for the inclusion criteria might affect the prognosis of the patients. The various new treatments have been introduced including molecularly-targeted therapy in recent years and further prospective studies are needed. Third, we could not evaluate histopathological correlations with imaging in patients who had not undergone lymphadenectomy. However, this population is diagnosed as stage IIICr of the FIGO 2018 classifications, and we have shown the prognostic utility of ^18^F-FDG PET compared with CT for this population.

## Conclusion

^18^F-FDG PET offers higher prognostic value than CT for patients with stage IIICr allocated by imaging, suggesting that ^18^F-FDG PET might be an optimal modality for use in clinical staging and for deciding diagnostic and therapeutic strategies.

### Supplementary Information

Supplementary Figure 1.

Supplementary Figure 2.

Supplementary Figure 3.

Supplementary Tables.

## Data Availability

The datasets used and/or analyzed during the current study are available from the corresponding author on reasonable request.

## Abbreviations

FIGO: The International Federation of Gynecology and Obstetrics
CT: Computed tomography
^18^F-FDG: ^18^F-fluorodeoxyglucose
PET: Positron emission tomography
MRI: Magnetic resonance imaging
OSEM: Ordered subset expectation maximization
3D: Three-dimensional
MR-AC: MR attenuation correction
SUV: Standardized uptake value
PFS: Progression-free survival
OS: Overall survival
