# Supplementary figures and images for: Prognostic value of 18F-FDG PET in uterine cervical cancer patients with stage IIICr allocated by imaging
Source: Sci Rep. 2023 Nov 1;13:18864. doi: 10.1038/s41598-023-46261-2 (PMC10620427; doi:10.1038/s41598-023-46261-2)

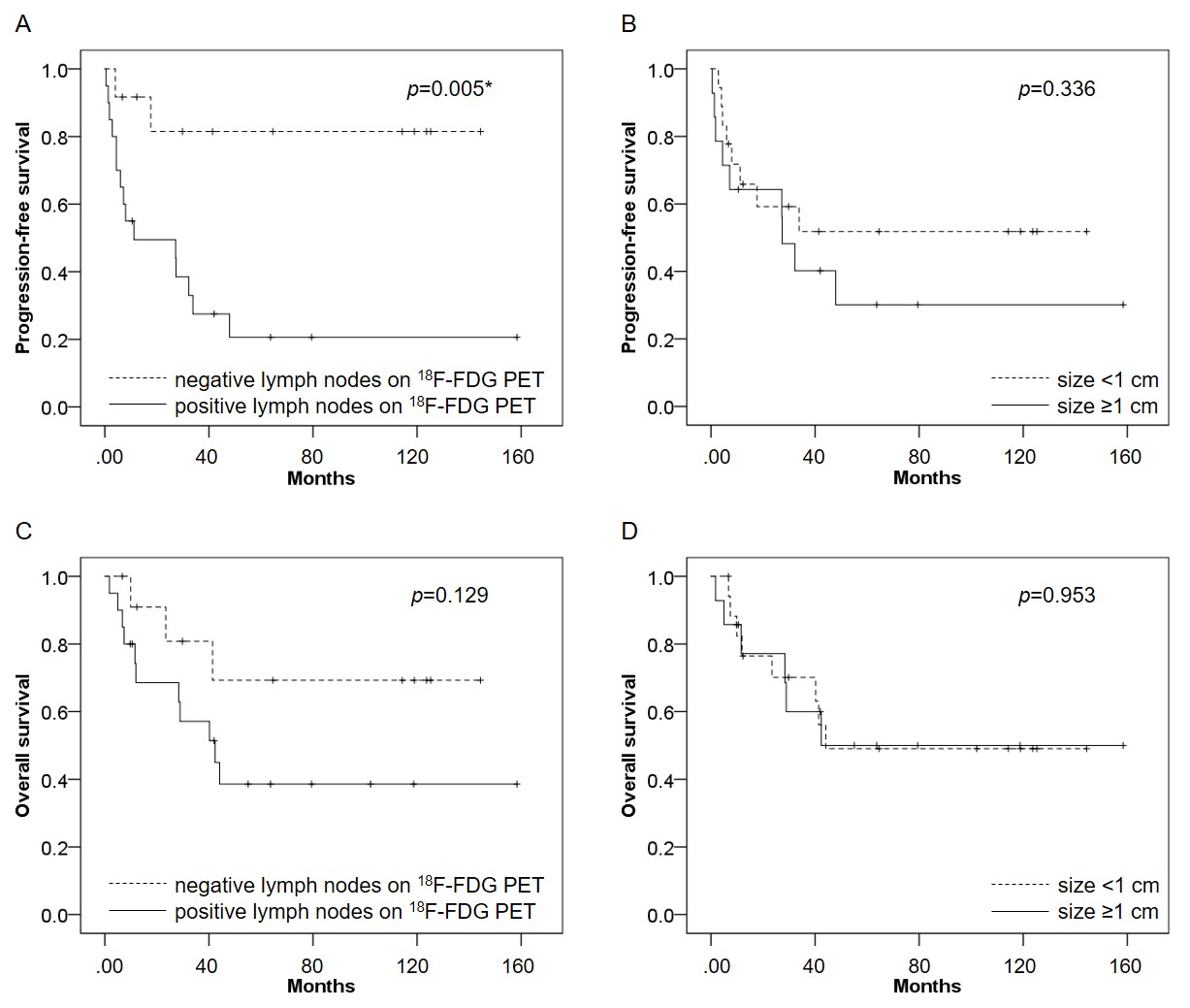

Supplement: Supplementary file 1 — Supplementary Figure 1. [file 41598_2023_46261_MOESM1_ESM.jpg]

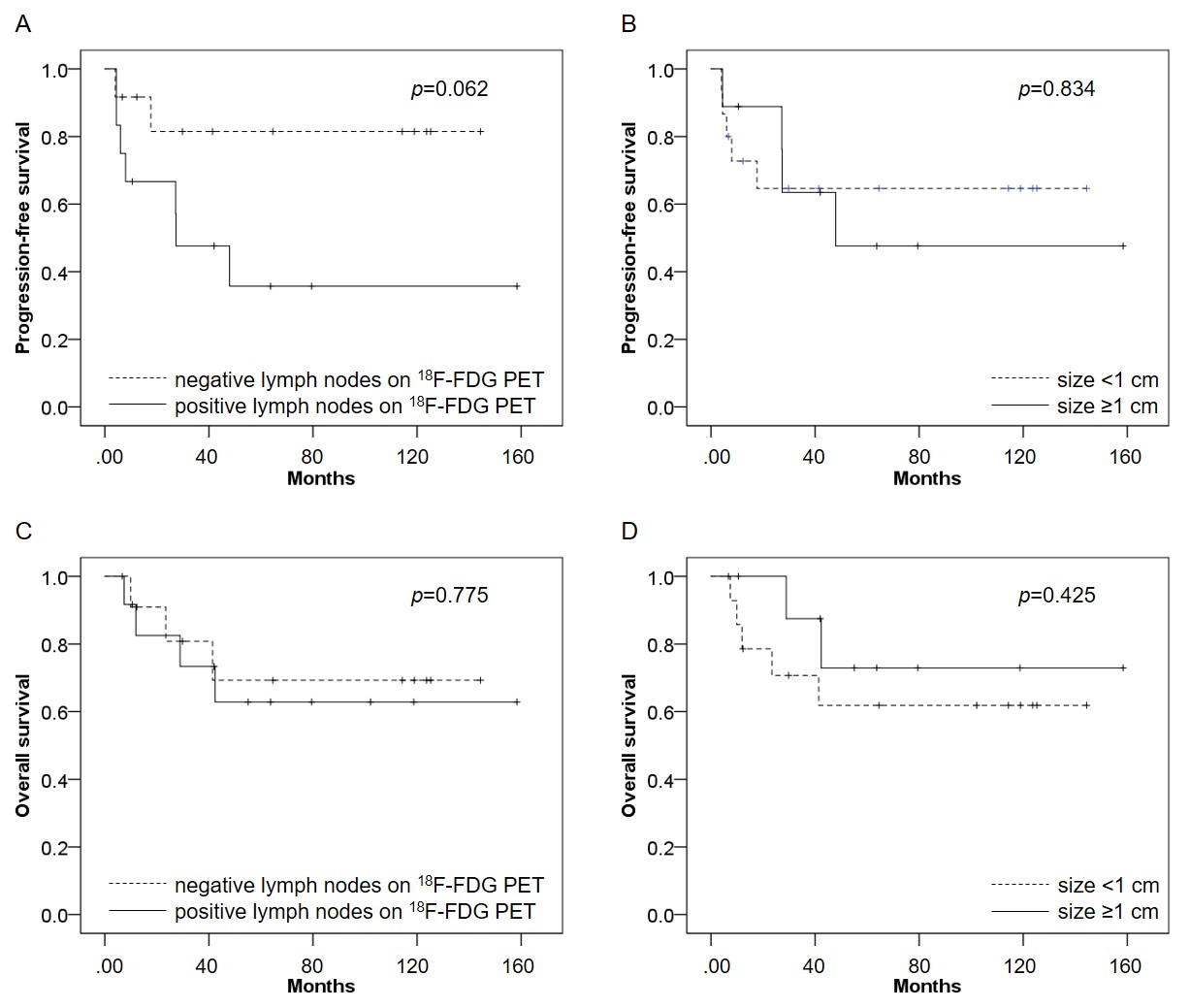

Supplement: Supplementary file 2 — Supplementary Figure 2. [file 41598_2023_46261_MOESM2_ESM.jpg]

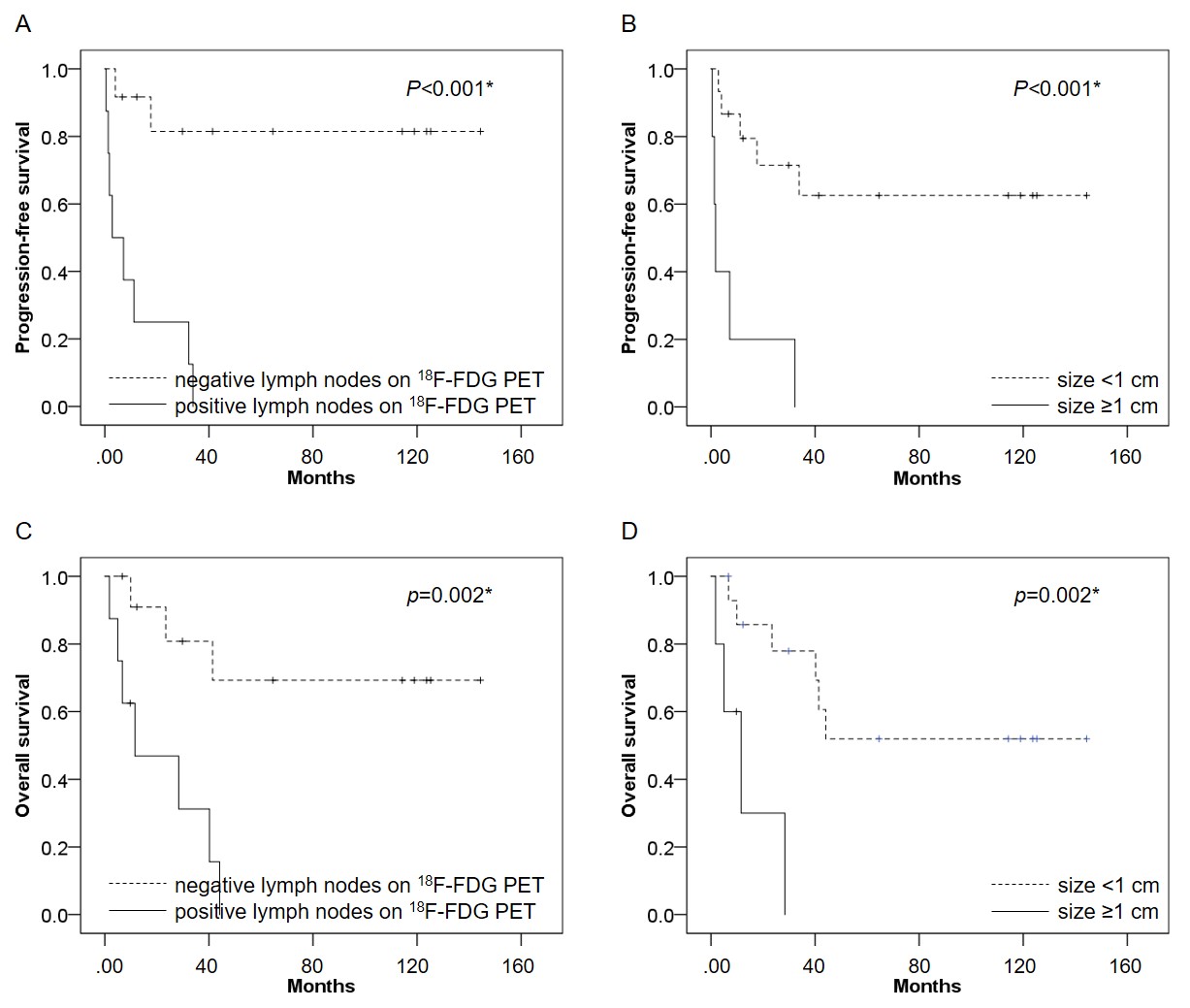

Supplement: Supplementary file 3 — Supplementary Figure 3. [file 41598_2023_46261_MOESM3_ESM.jpg]
