# Supplementary material for: Prognostic value of 18F-FDG PET in uterine cervical cancer patients with stage IIICr allocated by imaging
Source: Sci Rep. 2023 Nov 1;13:18864. doi: 10.1038/s41598-023-46261-2 (PMC10620427; doi:10.1038/s41598-023-46261-2)
Supplement: Supplementary file 4 — Supplementary Tables. [file 41598_2023_46261_MOESM4_ESM.docx]

Supplemental table 1. Patient and tumor characteristics based on FIGO stage

|  | FIGO stage | | | | | | |
| --- | --- | --- | --- | --- | --- | --- | --- |
|  | IIB | IIIA | IIIB | IIIC1r | IIIC2r | IVA | IVB |
| Imaging findings of lymph nodes |  |  |  |  |  |  |  |
| negative lymph nodes on ^18^F-FDG PET and size<1cm | 1 | 2 | 12 | 0 | 0 | 3 | 0 |
| positive lymph nodes on ^18^F-FDG PET and size<1cm | 0 | 0 | 0 | 6 | 3 | 0 | 2 |
| negative lymph nodes on ^18^F-FDG PET and size≥1cm | 0 | 0 | 0 | 0 | 0 | 0 | 0 |
| positive lymph nodes on ^18^F-FDG PET and size≥1cm | 0 | 0 | 0 | 11 | 9 | 1 | 5 |
| Combined chemotherapy |  |  |  |  |  |  |  |
| radiotherapy only | 1 | 1 | 5 | 2 | 6 | 2 | 2 |
| concurrent chemoradiotherapy | 0 | 1 | 7 | 15 | 6 | 2 | 5 |
| Combined brachtherapy |  |  |  |  |  |  |  |
| external beam radiation only | 0 | 1 | 1 | 3 | 4 | 3 | 6 |
| combined brachtherapy | 1 | 1 | 11 | 14 | 8 | 1 | 1 |
| Tumor progression |  |  |  |  |  |  |  |
| absence | 1 | 2 | 10 | 6 | 3 | 1 | 1 |
| presence | 0 | 0 | 2 | 11 | 9 | 3 | 6 |
| Death |  |  |  |  |  |  |  |
| absence | 1 | 1 | 9 | 11 | 3 | 1 | 3 |
| presence | 0 | 1 | 3 | 6 | 9 | 3 | 4 |

FIGO; The International Federation of Gynecology and Obstetrics ^18^F-FDG PET; ^18^F-fluorodeoxyglucose Positron Emission Tomography

Table 2. Prognostic factors for progression-free survival in 32 T3b patients with IIIB in 12 or IIICr in 20

| Variable | Univariate analysis |  | Multivariate analysis |  |
| --- | --- | --- | --- | --- |
|  | Hazard ratio (95%CI) | P | Hazard ratio (95%CI) | P |
| Age (y) | 1.02 (0.98–1.05) | 0.430 |  |  |
| Histopathologic type (adenocarcinoma) | 2.37 (0.68–8.29) | 0.176 |  |  |
| Concurrent chemoradiotherapy | 0.56 (0.20–1.55) | 0.264 |  |  |
| Brachytherapy | 0.34 (0.11–1.06) | 0.064 | 0.26 (0.08–0.89) | 0.032* |
| Positive lymph nodes on ^18^F-FDG PET | 6.42 (1.46–28.23) | 0.014* | 7.31 (1.62–33.00) | 0.010* |
| Size ≥1 cm | 1.59 (0.61–4.12) | 0.342 |  |  |

*Cox’s uni- and multivariate analysis

^18^F-FDG PET; ^18^F-fluorodeoxyglucose Positron Emission Tomography

Table 3. Prognostic factors for overall survival in 32 T3b patients with IIIB in 12 or IIICr in 20

| Variable | Univariate analysis |  | Multivariate analysis |  |
| --- | --- | --- | --- | --- |
|  | Hazard ratio (95%CI) | P | Hazard ratio (95%CI) | P |
| Age (y) | 1.02 (0.98–1.07) | 0.282 |  |  |
| Histopathologic type (adenocarcinoma) | 2.88 (0.79–10.42) | 0.108 |  |  |
| Concurrent chemoradiotherapy | 0.41 (0.14–1.22) | 0.109 |  |  |
| Brachytherapy | 0.24 (0.07–0.79) | 0.019* | 0.18 (0.05–0.67) | 0.011* |
| Positive lymph nodes on ^18^F-FDG PET | 2.60 (0.72–9.32) | 0.143 | 3.30 (0.86–12.68) | 0.083 |
| Size ≥1 cm | 1.03 (0.36–2.98) | 0.954 |  |  |

*Cox’s uni- and multivariate analysis

^18^F-FDG PET; ^18^F-fluorodeoxyglucose Positron Emission Tomography
